# Accuracies of univariate and multivariate genomic prediction models in African cassava

**DOI:** 10.1186/s12711-017-0361-y

**Published:** 2017-12-04

**Authors:** Uche Godfrey Okeke, Deniz Akdemir, Ismail Rabbi, Peter Kulakow, Jean-Luc Jannink

**Affiliations:** 1 Section of Plant Breeding and Genetics, School of Integrative Plant Sciences, College of Agriculture and Life Sciences, Cornell University, Ithaca, NY 14853 USA; 2 International Institute of Tropical Agriculture (IITA), PMB 5320, Oyo Road, Ibadan, Nigeria; 3 Robert W. Holley Center for Agriculture and Health, USDA-ARS, Tower Road, Ithaca, NY 14853-2901 USA

## Abstract

**Background:**

Genomic selection (GS) promises to accelerate genetic gain in plant breeding programs especially for crop species such as cassava that have long breeding cycles. Practically, to implement GS in cassava breeding, it is necessary to evaluate different GS models and to develop suitable models for an optimized breeding pipeline. In this paper, we compared (1) prediction accuracies from a single-trait (uT) and a multi-trait (MT) mixed model for a single-environment genetic evaluation (Scenario 1), and (2) accuracies from a compound symmetric multi-environment model (uE) parameterized as a univariate multi-kernel model to a multivariate (ME) multi-environment mixed model that accounts for genotype-by-environment interaction for multi-environment genetic evaluation (Scenario 2). For these analyses, we used 16 years of public cassava breeding data for six target cassava traits and a fivefold cross-validation scheme with 10-repeat cycles to assess model prediction accuracies.

**Results:**

In Scenario 1, the MT models had higher prediction accuracies than the uT models for all traits and locations analyzed, which amounted to on average a 40% improved prediction accuracy. For Scenario 2, we observed that the ME model had on average (across all locations and traits) a 12% improved prediction accuracy compared to the uE model.

**Conclusions:**

We recommend the use of multivariate mixed models (MT and ME) for cassava genetic evaluation. These models may be useful for other plant species.

## Background

Cassava (*Manihot esculenta* Crantz) [1] is a staple food for over 700 million people in Africa, South America and Asia [2]. Cassava also has immense industrial potential. White cassava starch is easy to extract and contains low levels of fat (~ 1.5%), protein (~ 0.6%) and phosphorus (~ 4%), which are desirable features for the food industry [3, 4]. Given the issues of climate change and fast-growing populations in countries that rely heavily on cassava, the need for rapid genetic improvement of cassava is critical. To enable this, genetic evaluation protocols based on best linear unbiased prediction (BLUP) analysis [5, 6] and selection on a merit index [7, 8] have been recommended to maximize gain from selection [9].

Genomic selection (GS) [10] offers crop species such as cassava a tremendous opportunity for accelerated genetic gains [11] by using whole-genome single nucleotide polymorphisms (SNPs) scored with methods such as genotyping-by-sequencing (GBS) [12]. These whole-genome SNPs could be sufficiently dense to be in linkage disequilibrium with most quantitative trait loci (QTL) that affect traits of interest. Using GS, selection is imposed at these QTL without actually identifying the QTL or the functional polymorphisms [10]. In addition, these SNPs will help to better track relatedness due to Mendelian sampling [13], which leads to improved selection accuracies especially when pedigree records are not available [14].

## GS models for plant genetic evaluation

Genetic evaluation [9] starts by accurately estimating the genetic value of an individual for a wide range of traits using its own performance records, progeny performance records, records from relatives, or a combination of the three [15]. Usually this estimation is carried out by using the univariate single environment one-step (uT) BLUP model [16] to obtain estimated breeding values (EBV) for one trait at a time. In plant and animal breeding, breeders usually select multiple traits at the same time that are often genetically correlated, with correlations that can range from weak to strong. The uT model for traits that are measured in a single environment assumes zero genetic and residual covariances between these traits such that information from other traits is not used when obtaining EBV of the evaluated individuals for the traits in the analysis. However, the optimal estimation procedure is to combine information from multiple trait records and obtain EBV using the multi-trait single environment one-step BLUP model (MT) [17, 18]. The MT model does not assume zero genetic and residual covariances but provides an estimate for these and also uses this information when obtaining individual EBV for the traits in the analysis. The MT model has several advantages over the uT model including:

1. Higher prediction accuracies for individual traits in the model because of more information (direct or indirect) and better connectedness of the data [19], especially when traits with varying heritabilities are analyzed jointly. This is true if the genetic correlations in the model are significant or substantial with low error correlations.
2. Simplified index selection because optimal weight factors for the total merit index are the economic weights [19].
3. Procedures for obtaining genetic and residual covariances and incorporating these in EBV estimates for across-location, -country or -region evaluations [20–22].

While MT models have clear advantages over uT models, they require the estimation of additional parameters (i.e., genetic and error covariances), which will affect accuracies of EBV. The number of additional parameters increases as the number of traits increases. For large models, many additional parameters can lead to convergence problems in the analysis. Lastly, an appreciable amount of data is required to get good estimates of these additional parameters.

In most plant breeding programs, genotypes are evaluated in multi-environment trials (MET) usually at advanced stages of breeding. The goal is to sample the influence on selection candidates of the range of environments for which varieties will be targeted. Addressing the problem of the analysis of MET brings into focus another potential use for MT models [23]. Here, phenotypes of the same trait, but measured at different locations are parameterized as different traits in the MT model [24], producing what we call a multivariate single-trait multi-environment BLUP (ME) model. Like the MT model, the ME model estimates genetic covariances of the same trait measured in multiple environments, which may lead to more accurate estimates of individual EBV for the trait in all the environments in which data are recorded. For the ME models used for modelling MET data, residual covariances are set to zero reflecting the assumption that no mechanism generates error covariances of a trait measured in different environments [20]. In contrast, the typical univariate BLUP model for modelling MET data, termed the univariate multi-environment one-step model (uE), fits a multi-kernel mixed model with the genotypic effect as one kernel and the genotype-by-environment (GxE) effect as the second kernel and maybe environment as the third kernel [25]. This model yields a GxE variance for a MET and individuals can be ranked on their performance in different locations. Different variants of the ME model have been used for modeling environment covariance structures in plant [26–29] and in animal breeding [30, 31]. Genetic covariances from the ME model offer a convenient tool to assess the impact of GxE on a trait and relate directly to the extent of GxE at all locations in the analysis. A low genetic correlation between EBV for a trait at different locations from the ME model indicates a high GxE impact on that trait [9, 32–35].

To select the GS model for a practical cassava breeding program, it is necessary to compare models that will be efficient at various stages of cassava breeding with MET data. Finally, fitting multivariate BLUP models is not trivial. Even with software that, in principle, can fit these models, model convergence is not guaranteed and may require several attempts [36–38] and univariate models may be more practical if the benefits of the multivariate models are not substantial.

The objectives of this paper are to (1) compare multi-trait (MT) and single trait (uT) mixed models for single environment data using cross-validation, and (2) compare the multivariate multi-environment (ME) model to a single-trait multi-environment (uE) model using cross-validation and assessing the GxE impact on the traits analyzed via genetic covariances from the ME model fit.

## Methods

### Cassava phenotype data

We used historical phenotype data from different trials that were conducted for the cassava breeding program at the International Institute of Tropical Agriculture (IITA), Ibadan, Nigeria. The genetic gain population represents a collection of clones selected from the 1970s to 2007 within this program [39, 40]. Some of these clones are West African landraces and some are of East African origin. Clones in the genetic gain population have gone through advanced stages of the cassava breeding process up to on-farm variety testing trials. The data used in our analysis include data that were collected on clonal evaluation trials (CET), which are augmented design trials with typically two known checks and unreplicated plots with five plants. These data were collected from three target locations in Nigeria: Ibadan (7.40°N, 3.90°E), Mokwa (9.3°N, 5.0°E), and Ubiaja (6.66°N, 6.38°E). These locations represent regions, which encompass about 35% of the cassava production in Nigeria. Datasets were collected from 2000 to 2015 and included trials with most of the 739 clones of the genetic gain population. Six target agronomic traits were used in the analysis including seedling vigor (VIGOR), number of storage roots per plot at harvest (RTNO), fresh weight of harvested roots expressed in tons per hectare (T/ha) (FYLD), percent dry matter (DM) of storage roots, which measures root dry weight as the % of the root fresh weight, plot mean cassava mosaic disease severity (MCMDS), which is rated on a scale from 1 (no symptoms) to 5 (extremely severe), and plot mean cassava green mite (MCGM) severity, which is rated on a scale from 1 (no symptoms) to 5 (extremely severe). Cassava mosaic disease is caused by a *Begomovirus* that belongs to the Geminiviridae family, and is carried and transmitted by the whitefly *Bemisia tabaci*. The cassava green mite is *Mononychellus tanajoa* [41]. These traits are target traits used in the selection index for selection decisions in the IITA cassava breeding program. Phenotype data metrics are in Table 1. All trait records were plot averages for both clonal accessions and checks. All checks were included in the analysis.

**Table 1:** Cassava phenotype means and standard deviations (in brackets) at three locations: Ubiaja, Mokwa, and Ibadan

	Ubiaja	Mokwa	Ibadan
Number of records	7806	5345	5579
Number of clones	739	573	691
VIGOR (1–3)	6.51 (1.12)	6.52 (0.93)	6.11 (1.23)
RTNO (No./plot)	31.71 (17.30)	37.05 (21.76)	37.87 (23.91)
FYLD (T/ha)	12.61 (7.70)	16.51 (9.54)	15.84 (10.72)
DM (%)	31.95 (6.42)	29.01 (6.38)	30.8 (6.79)
MCMDS (1–5)	1.59 (0.93)	1.21 (0.57)	2.14 (1.01)
MCGM (1–5)	3.56 (0.97)	2.99 (0.67)	3.00 (0.85)

*VIGOR* seedling vigor, *RTNO* number of storage roots per plot at harvest, *FYLD* fresh weight of harvested roots in tons per hectare, *DM* percentage dry matter of storage roots, *MCMDS* plot mean cassava mosaic disease severity, and *MCGM* plot mean cassava green mites severity

### Cassava genotyping data

DNA from 739 clones from the 2013 genetic gain trial at IITA was extracted by using DNeasy Plant Mini kits (Qiagen) and was quantified using PicoGreen. Genotyping-by-sequencing (GBS) was used for genotyping [12] these clones. Six 95-plex and one 75-plex *Ape*KI libraries were constructed and sequenced on Illumina HiSeq, one lane per library. SNPs were called from the sequence data using the TASSEL pipeline version 4.0 [42], using an alignment to the *Manihot esculenta* version 6 reference genome [43]. Average sequencing depth for polymorphic loci was 5×. Individuals with more than 80% and SNPs with more than 60% missing calls were removed. SNP genotyping data were converted to numeric genotypes (0, 1, 2) and missing genotyping data were imputed using a LASSO regression method (Ariel Chan, personal communication, 2014) that was implemented using the R glmnet package [44]. The resulting dataset was rounded to obtain numerical genotypes (0, 1, 2) and consisted of 183,201 SNPs scored in 739 clones.

### Statistical analysis

We structured the cassava phenotype data described above into two types of data that are commonly used in most plant breeding programs. The first set was achieved by pooling data from multiple years at specific locations (multi-year trials data). We termed this scenario the single-environment genetic evaluation (Scenario 1). The resulting prediction accuracies from this dataset were assessed for the three locations. The second scenario was achieved by using data from multiple locations and years (MET) but, in this case, location-specific information was extracted by modeling GxE interactions. We termed this scenario the multi-environment genetic evaluation (Scenario 2) and its goal was to assess the impact of GxE and determine the best way to fit it while also using information from correlated environments.

### Pseudo-true genetic values for prediction accuracy computations

To validate the models in this study, first we defined a univariate single-trait mixed model for each trait at each location separately (to preserve the variation embedded in each location) using an identity covariance matrix among clone effects, which assumes no relationship among the clones. The univariate mixed model was as follows:

1 $$ {\mathbf{y}} = {\mathbf{Xb}} + {\mathbf{Zu}} + {\mathbf{e}}, $$

with ${\mathbf{u}} \sim N\left( {0,\upsigma_{\text{u}}^{2} {\mathbf{I}}} \right)$ and ${\mathbf{e}} \sim N\left( {0,\upsigma^{2} {\mathbf{I}}} \right)$, where ${\mathbf{y}}$ is a vector of observations, ${\mathbf{b}}$ is a vector of fixed effects with the design matrix ${\mathbf{X}}$ (relating observations to fixed effects, in this case including grand mean and a nested effect of trial-within-year and the ratio of plants harvested to number planted); ${\mathbf{u}}$ is a vector of clonal genetic effects with the design matrix ${\mathbf{Z}}$ (relating observations to clones). This model was fit using the *lmer* function in the R lme4 package [45] and resulting BLUP values ($\hat{u}$), which we refer to as estimated genotypic values (EGV), were used as pseudo-true genetic effects to compute prediction accuracy, as commonly used in the plant breeding literature [41, 46, 47].

### GS models for Scenario 1

We defined two mixed models that were fitted as follows.

#### The single-trait mixed model (uT)

2 $$ {\mathbf{y}} = {\mathbf{X{\varvec{\upbeta}} }} + {\mathbf{Zu}} + {\mathbf{e}}, $$

with ${\mathbf{u}} \sim N\left( {0,\upsigma_{\text{u}}^{2} {\mathbf{K}}} \right)$ and ${\mathbf{e}} \sim N\left( {0,\upsigma^{2} {\mathbf{I}}} \right)$, where ${\mathbf{y}}$ is the response vector of a trait for a given location, ${\varvec{\upbeta}}$ is the vector of fixed effects with the design matrix ${\mathbf{X}}$ (with components as in Model 1 above for each location and trait); ${\mathbf{u}}$ is the vector of random additive genomic effects with the design matrix ${\mathbf{Z}}$ (relating trait values to clones) and ${\mathbf{K}}$ is the additive genomic relationship matrix generated from SNPs as in method 1 of VanRaden [48] implemented in preGSf90 [49].

#### The multitrait mixed model

3 $$ {\mathbf{y}} = {\mathbf{X{\varvec{\upbeta}} }} + {\mathbf{Zu}} + {\mathbf{e}}, $$

where ${\mathbf{y}} = ({\mathbf{y}}_{1}^{{\prime }} ,{\mathbf{y}}_{2}^{{\prime }} ,{\mathbf{y}}_{3}^{{\prime }} , \ldots , {\mathbf{y}}_{\text{d}}^{{\prime }} )^{ '}$, ${\mathbf{u}} = ({\mathbf{u}}_{1}^{{\prime }} ,{\mathbf{u}}_{2}^{{\prime }} , \ldots , {\mathbf{u}}_{\text{d}}^{{\prime }} )^{ '}$ and ${\mathbf{e}} = ({\mathbf{e}}_{1}^{{\prime }} ,{\mathbf{e}}_{2}^{{\prime }} ,{\mathbf{e}}_{3}^{{\prime }} , \ldots , {\mathbf{e}}_{\text{d}}^{{\prime }} )^{ '}$, and ${\mathbf{y}}$ is the response vector of $d$ traits (six core traits described above), ${\mathbf{X}}$ is a design matrix for fixed effects ${\varvec{\upbeta}}$ (with components as in Model 1 above for each location and trait) and ${\mathbf{Z}}$ is a design matrix for random genetic effects ${\mathbf{u}}$. Following a multivariate normal distribution ($N_{m}$), the marginal density of ${\mathbf{y}}$ is:

4 $$ ({\mathbf{y}}  |  {\varvec{\upbeta}}, {\mathbf{R}}, {\mathbf{G}}) \sim {\text{N}}_{\text{m}} \left( {{\mathbf{X{\varvec{\upbeta}}}}, {\mathbf{V}}} \right) $$

and $$ {\mathbf{V}} = {\mathbf{Z}}\left( {{\mathbf{G}} \otimes {\mathbf{K}}} \right){\mathbf{Z}}^{\text{T}} + {\mathbf{R}} \otimes {\mathbf{I}}) $$.

The matrices ${\mathbf{G}}$ and ${\mathbf{R}}$ are $d \times d$ symmetric unstructured genomic and error covariance matrices respectively, ${\mathbf{K}}$ remains the additive genomic relationship matrix for $n$ clones generated from SNPs as above, ${\mathbf{I}}$ is an identity matrix.

Models (2) and (3) were fitted separately for each location Ubiaja, Mokwa and Ibadan, respectively, allowing the error (co)variances associated with these locations to be distinct. Note also that, in these models, genotype-by-location effects are confounded with the main genotype effects such that variance components may change between locations. The effects of years and trials were fixed because emphasis was on location effects since these locations represented different production regions and we sought to capture consistent effects of these locations. In contrast, year effects are variable and by definition not consistent. Also following practice in cassava breeding [46, 47], multiple observations of one clone were not considered as repeated measures. Although these subjects were clones, data were collected from distinct individuals and thus they are independent. Hence, these measurements were treated as samples of clones and should lead to better precision in the prediction of breeding values.

### GS models for Scenario 2

We also defined two mixed models with the aim of modeling genotype-by-environment interaction effects as follows.

#### The compound symmetric multi-environment model (uE)

Here, we describe the uE model, first how it is fit and then we show its compound symmetry structure. The model is as follows:

5A $$ {\mathbf{y}} = {\mathbf{X{\varvec{\upbeta}} }} + {\mathbf{Z}}_{1} {\mathbf{u}} + {\mathbf{Z}}_{2} {\mathbf{w}} + {\mathbf{e}}, $$

with $$ {\mathbf{y}} = ({\mathbf{y^{\prime}}}_{\text{a}} ,{\mathbf{y^{\prime}}}_{\text{b}} ,{\mathbf{y^{\prime}}}_{\text{c}} )^{ '} $$, $$ {\mathbf{e}} = ({\mathbf{e^{\prime}}}_{\text{a}} ,{\mathbf{e^{\prime}}}_{\text{b}} ,{\mathbf{e^{\prime}}}_{\text{c}} )^{ '} $$,

$$ {\mathbf{u}} \sim {\text{N}}\left( {0,  \upsigma_{\text{u}}^{2} {\mathbf{K}}} \right),\;{\mathbf{w}} \sim {\text{N}}\left( {0,  \upsigma_{\text{w}}^{2} {\mathbf{I}}_{3} \otimes {\mathbf{K}}} \right),\;{\mathbf{e}} \sim {\text{N}}\left( {0,\upsigma_{\text{e}}^{2} {\mathbf{I}}} \right) $$

where ${\mathbf{y}}$ is the vector of a trait at locations ${\text{a}}$, ${\text{b}}$ and ${\text{c}}$ (corresponding to Ubiaja, Mokwa and Ibadan), ${\varvec{\upbeta}}$ is the vector of fixed effects with the design matrix ${\mathbf{X}}$ (relating observations to fixed effects as in Model 1); ${\mathbf{u}}$ is the vector of random additive genomic effects with the design matrix ${\mathbf{Z}}_{1}$ (relating trait values to clones), ${\mathbf{w}}$ is the vector of random clone-by-location interaction effects with the design matrix ${\mathbf{Z}}_{2}$, which is $diag\left( {{\mathbf{Z}}_{\text{a}} ,\varvec{ }{\mathbf{Z}}_{\text{b}} ,\varvec{ }{\mathbf{Z}}_{\text{c}} } \right)$ that relates records to clones in locations ${\text{a}}$, ${\text{b}}$ and ${\text{c}}$, respectively. For the ${\text{c}}$th location, a column of ${\mathbf{Z}}_{\text{c}}$ may be all 0s if the clone represented by the column was not evaluated in that location. $\upsigma_{\text{u}}^{2} {\mathbf{K}}$ is the additive genomic relationship matrix generated from SNPs as above, ${\mathbf{I}}$ is an identity matrix and ${\mathbf{I}}_{3}$ is a $3 \times 3\varvec{ }$ identity matrix. In this model, the genomic value of a clone for the ${\text{c}}$th location was estimated as ${\hat{\mathbf{u}}} + \widehat{{{\mathbf{w}}_{\text{c}} }}$. A more complete account of the error terms would have included clone-by-year and clone-by-location-by-year terms in the model. While such a model would have characterized the error in more detail, we believe that the obtained improvement of within-location estimation would have been marginal. Model 5 implies a compound symmetry structure [50] as described below.

#### The uE model defined as a compound symmetry (CS) covariance structure model

Using the same symbols as above, we defined the uE model with a CS covariance structure as:

5B $$ {\mathbf{y}} = {\mathbf{X{\varvec{\upbeta}}}} + {\mathbf{Z}}_{2} {\mathbf{w}} + {\mathbf{e}}, $$

with $$ {\mathbf{y}} = ({\mathbf{y^{\prime}}}_{\text{a}} ,{\mathbf{y^{\prime}}}_{\text{b}} ,{\mathbf{y^{\prime}}}_{\text{c}} )^{ '} $$ and $$ {\mathbf{e}} = ({\mathbf{e^{\prime}}}_{\text{a}} ,{\mathbf{e^{\prime}}}_{\text{b}} ,{\mathbf{e^{\prime}}}_{\text{c}} )^{ '} $$,

$$ {\mathbf{w}} \sim N\left( {0, \left( {\upsigma_{\text{u}}^{2} +\upsigma_{\text{w}}^{2} } \right){\varvec{\Phi}} \otimes {\mathbf{K}}} \right), $$

$$ {\varvec{\Phi}} = \left[ {\begin{array}{*{20}c} 1 &\uprho &\uprho \\\uprho & 1 &\uprho \\\uprho &\uprho & 1 \\ \end{array} } \right]\quad {\text{with}}\quad\uprho = \frac{{\sigma_{u}^{2} }}{{\sigma_{u}^{2} + \sigma_{w}^{2} }}, $$

$$ \varvec{V} = (\upsigma_{\text{u}}^{2} +\upsigma_{\text{w}}^{2} ) {\mathbf{Z}}_{2} \left( {{\varvec{\Phi}} \otimes {\mathbf{K}}} \right){\mathbf{Z}}_{2}^{'} +\upsigma_{\text{e}}^{2} {\mathbf{I}}. $$

The genomic effect from this CS model for the $$ {\text{c}} $$th location $$ \widehat{{{\mathbf{w}}_{c} }} $$ is equal to $$ {\mathbf{\overset{\lower0.5em\hbox{$\smash{\scriptscriptstyle\frown}$}}{u} }} + \widehat{{{\mathbf{w}}_{c} }} $$ from the uE model. The $$ {\mathbf{Z}}_{2} $$ matrix is the same as in the uE model. Compared to the ME model described below, which replaces $$ {\varvec{\Phi}} $$ with an unstructured covariance matrix with nine parameters (six for genetic and three for error (co)variances, respectively), the CS model has three parameters including $$ \upsigma_{{{\text{u}} + {\text{w}}}}^{2} $$ (equivalent to $$ \upsigma_{\text{u}}^{2} +\upsigma_{\text{w}}^{2} $$ in the uE model), $$ \upsigma_{\text{e}}^{2} $$ and $$ \uprho $$. For any trait for which the CS covariance structure best fits the data, it is expected that model uE will provide more accurate GEBV than the ME model, which will overfit the data. Furthermore, the uE model defined here assumes a homogeneous variance across locations $$ {\text{a}} $$, $$ {\text{b}} $$ and $$ {\text{c}} $$. Although a CS model with heterogeneous variances can be fit, this was not the case for the uE model. This assumption is incorrect if there are significant heterogeneous variances across these locations. In such a case, the ME model should provide more accurate breeding values.

### The multivariate multi-environment (ME) model

We fit the ME model in a single-step procedure using the following model:

6 $$ {\mathbf{y}} = {\mathbf{X{\varvec{\upbeta}} }} + {\mathbf{Zu}} + {\mathbf{e}}, $$

with ${\mathbf{y}} = ({\mathbf{y^{\prime}}}_{\text{a}} ,{\mathbf{y^{\prime}}}_{\text{b}} ,{\mathbf{y^{\prime}}}_{\text{c}} )^{ '}$, ${\mathbf{u}} = ({\mathbf{u^{\prime}}}_{\text{a}} ,{\mathbf{u^{\prime}}}_{\text{b}} , {\mathbf{u^{\prime}}}_{\text{c}} )^{ '}$ and ${\mathbf{e}} = ({\mathbf{e^{\prime}}}_{\text{a}} ,{\mathbf{e^{\prime}}}_{\text{b}} ,{\mathbf{e^{\prime}}}_{\text{c}} )^{ '}$, where ${\mathbf{y}}$ is the vector of a trait in locations ${\text{a}}$, ${\text{b}}$ and ${\text{c}}$ (corresponding to Ubiaja, Mokwa and Ibadan) recorded for $n$ clones, the ${\mathbf{X}}$ and ${\mathbf{Z}}$ design matrices are block diagonal matrices represented as $diag\left( {{\mathbf{X}}_{\text{a}} ,\varvec{ }{\mathbf{X}}_{\text{b}} ,\varvec{ }{\mathbf{X}}_{\text{c}} } \right)$ and $diag\left( {{\mathbf{Z}}_{\text{a}} ,\varvec{ }{\mathbf{Z}}_{\text{b}} ,\varvec{ }{\mathbf{Z}}_{\text{c}} } \right)$, respectively allowing for missing clones and observations. ${\mathbf{X}}$ is a design matrix for fixed effects ${\varvec{\upbeta}}$ (with components as in Model 1) and ${\mathbf{Z}}$ is a design matrix for random genomic effects ${\mathbf{u}}$. Following a multivariate normal distribution ($N_{m}$), the marginal density of ${\mathbf{y}}$ is:

7 $$ ({\mathbf{y}}  |  {\varvec{\upbeta}}, {\mathbf{R}}, {\mathbf{G}}) \sim {\text{N}}_{\text{m}} \left( {{\mathbf{X }\varvec{\upbeta}}, {\mathbf{V}}} \right), $$

and $$ {\mathbf{V}} = {\mathbf{Z}}\left( {{\mathbf{G}} \otimes {\mathbf{K}}} \right){\mathbf{Z}}^{\text{T}} + {\mathbf{R}} \otimes {\mathbf{I}}) $$.

Given that $d$ is the number of locations being analyzed, ${\mathbf{G}}$ is a $d \times d$ symmetric and unstructured genomic covariance matrix, while ${\mathbf{R}}$ is a $d$-dimensional diagonal error covariance matrix, ${\mathbf{K}}$ remains the additive genomic relationship matrix for $n$ clones generated from SNPs as above, and ${\mathbf{I}}$ is an identity matrix. In this model, the error covariance matrix ${\mathbf{R}}$ is diagonal, thus allowing heterogeneous variances for a trait at different locations but the covariances are fixed to zero following the assumption that no mechanism generates error covariances of a trait measured in multiple environments.

Estimation of the parameters in Models (2), (3), (5) and (6) were performed using the average information (AI) REML procedure implemented in the *airemlf90* program [49] from which the best linear unbiased estimator (BLUE) of fixed effects and the BLUP of random effects were obtained by solving the mixed model equations (MME) [5, 6]. Custom R-scripts were used for cross-validation.

### Comparison of prediction accuracies

We used a fivefold cross-validation scheme with 10 repeats for comparisons between the univariate and multivariate models. We used the same folds for the models in each scenario. Hereafter, we refer to predicted BLUP or genomic effects from these models as genomic EBV (GEBV). Prediction accuracies were calculated as a correlation of the validation fold GEBV to their corresponding EGV.

## Results

### Scenario 1: MT versus uT model

In Scenario 1, we observed that the prediction accuracies of the MT model were higher than those of the uT models for most traits and locations in our analysis (Table 2). On average (across traits and locations), the MT model yielded prediction accuracies that were 59% higher for VIGOR, 43% for RTNO, 27% for DM, 40% for MCMDS, 55% for FYLD and 18% for MCGM compared to the uT model. Averaged across traits and locations, the MT models were 40% more accurate than the uT models.

**Table 2:** Cross-validation prediction accuracies for GS models in Scenarios 1 and 2

Single-trait single environment (uT)				Multi-trait (MT)		
Trait	Ubiaja	Mokwa	Ibadan	Ubiaja	Mokwa	Ibadan
GS Scenario 1						
VIGOR	0.24 (0.02)	0.16 (0.03)	0.42 (0.02)	0.41 (0.02)	0.31 (0.03)	0.58 (0.01)
RTNO	0.32 (0.02)	0.17 (0.02)	0.37 (0.02)	0.46 (0.02)	0.24 (0.03)	0.53 (0.02)
DM	0.60 (0.01)	0.33 (0.02)	0.51 (0.01)	0.72 (0.01)	0.46 (0.02)	0.64 (0.02)
MCMDS	0.49 (0.01)	0.37 (0.03)	0.59 (0.01)	0.69 (0.02)	0.60 (0.04)	0.74 (0.01)
FYLD	0.41 (0.02)	0.11 (0.03)	0.40 (0.01)	0.58 (0.02)	0.30 (0.03)	0.55 (0.02)
MCGM	0.38 (0.01)	0.50 (0.02)	0.58 (0.01)	0.48 (0.01)	0.56 (0.02)	0.69 (0.01)

Single-trait multi-environment (uE)				Multi-environment (ME)		
Trait	Ubiaja	Mokwa	Ibadan	Ubiaja	Mokwa	Ibadan
GS Scenario 2						
VIGOR	0.22 (0.01)	0.10 (0.01)	0.37 (0.01)	0.24 (0.01)	0.12 (0.02)	0.32 (0.01)
RTNO	0.29 (0.01)	0.11 (0.01)	0.34 (0.01)	0.27 (0.02)	0.13 (0.01)	0.34 (0.02)
DM	0.49 (0.01)	0.20 (0.02)	0.40 (0.01)	0.60 (0.01)	0.35 (0.01)	0.50 (0.01)
MCMDS	0.40 (0.01)	0.23 (0.01)	0.53 (0.01)	0.48 (0.01)	0.39 (0.02)	0.57 (0.01)
FYLD	0.38 (0.01)	0.10 (0.02)	0.35 (0.01)	0.37 (0.01)	0.12 (0.03)	0.36 (0.02)
MCGM	0.31 (0.01)	0.48 (0.01)	0.56 (0.01)	0.38 (0.02)	0.47 (0.01)	0.55 (0.01)

Prediction accuracies for MT and uT models (GS Scenario 1) and for ME and uE models (GS Scenario 2)

The numbers in brackets are standard deviations for cross-validation repeat cycles

*VIGOR* seedling vigor, *RTNO* number of storage roots per plot at harvest, *FYLD* fresh weight of harvested roots in tons per hectare, *DM* percentage dry matter of storage roots, *MCMDS* plot mean cassava mosaic disease severity, *MCGM* plot mean cassava green mites severity

### Scenario 2: ME versus uE model

In Scenario 2, we observed different patterns of prediction accuracies of the uE and ME models. The ME model yielded higher prediction accuracies for DM and MCMDS at all locations. On average (across locations), the uE model resulted in prediction accuracies that were 2% better for VIGOR and 1% for RTNO, while the ME model resulted in prediction accuracies that were 32% better for DM, 24% for MCMDS, 5% for FYLD, and 4% for MCGM. Prediction accuracy of the ME model was 12% higher than that of the uE model averaged across all traits and locations in the model. Trait correlations from the ME model representing the expected correlated responses to selection ranged from 0.21 to 0.66 for VIGOR, 0.36 to 0.54 for RTNO, 0.57 to 0.81 for DM, 0.68 to 0.87 for MCMDS, 0.31 to 0.52 for FYLD and 0.24 to 0.53 for MCGM. Thus, genetic effects for MCMDS and DM were more consistent across locations than those for the other traits.

## Discussion

### Scenario 1: MT versus uT model

Some studies reported comparisons between MT and uT genomic prediction models using simulated data or real datasets [51–53]. Based on simulated datasets, Guo et al. [53] and Calus et al. [52] reported similar accuracies between MT and uT models with accuracies of the MT models for lowly heritable traits being slightly higher when the genomic correlations between the traits increased. Using Holstein and Jersey breed datasets from the US Dairy National evaluation program, VanRaden et al. [51] also reported similar accuracies between MT and uT models for all the traits analyzed. However, for several traits, they obtained accuracies that were slightly higher with the uT model than with the MT model. For highly heritable traits and especially if complete phenotypic data are available for these, accuracies obtained by the MT model are not clearly better than those obtained by the uT model [53]. Improvement in prediction accuracies with the MT model is accrued mostly for lowly heritable traits when they are analyzed jointly with highly heritable traits that have medium to high genetic correlations and low residual correlations [52, 53]. Our results were consistent with those of other studies [52, 53] since, in our analysis, our MT model yielded higher accuracies for most traits and locations and resulted from the joint analysis of low and high heritability traits. Most of the genetic correlations between traits at all locations in the MT models were significant (substantial) with low error correlations (not shown). These contributed to the increased prediction accuracies observed for the MT models compared to those of the uT models. Substantial increases in prediction accuracies of the MT models were observed for VIGOR, RTNO and FYLD, which had mostly moderate to high genetic correlations with other traits at all locations although their heritabilities were mostly low.

For parental selections in specific locations, we recommend the use of MT models but to confirm this conclusion, further studies on the selection gains obtained by applying these models are necessary.

### Scenario 2: ME versus uE model

Comparisons between different ME and uE genomic prediction models have been reported in plant breeding literature [54–56]. Burgueno et al. [29] conducted extensive modeling for multi-environment trials using pedigree and genomic markers and incorporated many covariance structures including diagonal, factor analytic (FA), identity and unstructured covariances for both the genomic and error components in their models. They observed that prediction accuracies of a genomic ME model with a diagonal genomic covariance structure and a diagonal error covariance structure (ME_D–D_) were higher than that of a genomic ME model with a FA genomic covariance structure and diagonal error covariance (ME_FA-D_) for most of the locations measured in their analysis based on a cross-validation scheme (CV1) [29]. This ME_D–D_ is a univariate model with fewer parameters but it can be compared to our uE model. Although the uE model in our study assumed identical genomic and error variances for all locations analyzed, total phenotypic variance was partitioned into direct clonal genomic, clone-by-location interaction and error variance components. Hence, effects due to clones and clone-by-location interaction were combined to generate location-specific GEBV, which can be compared to location-specific GEBV obtained in the ME_D–D_ model. Our results were in line with this study for the traits VIGOR and RTNO at all locations with the uE model yielding higher prediction accuracies than the ME models and differed for the traits DM and MCMDS at all locations with the ME models yielding higher prediction accuracies. However, on average across locations and traits, prediction accuracies of the ME models were higher.

To further understand the strength of the impact of GxE interaction on the cassava core traits analyzed in our study, we used information from the proportion of total variation explained by clone and clone-by-location effects from the uE model (Table 3). From the total variation explained by SNPs, the effect of clone-by-location interaction was approximately 30% for VIGOR, 48% for RTNO, 12% for DM, 15% for MCMDS, 56% for FYLD and 46% for MCGM. These proportions show strong clone-by-location interactions for FYLD, RTNO, MCGM and VIGOR but weak interactions for DM and MCMDS. In addition, the genetic correlations between the three locations for DM and MCMDS were relatively high ranging from 57 to 81 and 68 to 87%, respectively (Table 4), supporting our findings. These high correlations revealed that cassava DM and MCMDS were repeatable across the locations in our study, which suggests that genotypes selected for these traits will perform comparably across locations. From the genetic correlations in Table 4, improvement for RTNO and FYLD at Ubiaja will result in a correlated response of about 50% for these traits at Mokwa and about 35% at Ibadan. The low predicted correlated responses confirm that the environment had a higher impact on RTNO, FYLD, VIGOR and MCGM, thus improving these traits is more challenging. This makes a case for decentralized breeding especially for yield component traits. Breeding for good varieties that combine these core traits may be targeted towards specific locations or groups of locations with specific genotypes selected for these locations.

**Table 3:** Proportion of explained variance by clone and clone-by-location effects

Trait	Variance explained by effect (%)	
	Clone-by-location	Clone
VIGOR	4.52	10.70
RTNO	8.70	9.54
DM	4.59	33.52
MCMDS	10.00	58.90
FYLD	13.01	10.11
MCGM	7.00	8.33

These estimates are from effects based on genotyping by sequencing markers from the uE model

*VIGOR* seedling vigor, *RTNO* number of storage roots per plot at harvest, *FYLD* fresh weight of harvested roots in tons per hectare, *DM* percentage dry matter of storage roots, *MCMDS* plot mean cassava mosaic disease severity, *MCGM* plot mean cassava green mites severity

**Table 4:** Genetic correlations from the multi-environment analysis

Trait	Location	Ubiaja	Mokwa
VIGOR	Mokwa	0.39 (0.02)	
	Ibadan	0.66 (0.02)	0.21 (0.03)
RTNO	Mokwa	0.54 (0.08)	
	Ibadan	0.36 (0.08)	0.38 (0.08)
DM	Mokwa	0.57 (0.00)	
	Ibadan	0.81 (0.00)	0.77 (0.00)
MCMDS	Mokwa	0.80 (0.03)	
	Ibadan	0.87 (0.05)	0.68 (0.04)
FYLD	Mokwa	0.52 (0.02)	
	Ibadan	0.31 (0.02)	0.33 (0.04)
MCGM	Mokwa	0.34 (0.01)	
	Ibadan	0.24 (0.01)	0.53 (0.01)

The genetic correlations are from the ME model (standard error of estimates in brackets)

*VIGOR* seedling vigor, *RTNO* number of storage roots per plot at harvest, *FYLD* fresh weight of harvested roots in tons per hectare, *DM* percentage dry matter of storage roots, *MCMDS* plot mean cassava mosaic disease severity, *MCGM* plot mean cassava green mites severity

The ME model exploits the positive genomic correlations captured in its ${\mathbf{G}}$ matrix for prediction. The major difference between the prediction accuracies obtained by the ME and uE models were mainly due to the fact that the ME model accounted for genetic covariances when generating GEBV since genetic variances from both models were similar. Genetic covariances from the ME models are a reflection of the GxE interactions for the trait of interest and ME breeding values capture both additive genotypic and additive genotype-by-environment effects. However, the lack of information from between-trait correlations (which are captured by MT models) in ME breeding values represents a challenge when selection decisions based on information from the interconnection between multiple trait and multiple location data are required. The interconnection between these data may be useful for understanding GxE and for selection on traits that are highly influenced by the environment. Therefore, there is an opportunity for interconnection between information from a valuable single environment and MET data which are readily available in plant breeding programs.

Another potential use of the ME models is for clustering environments into target populations of environments (TPE). If correlated responses to selection of target traits are similar for certain locations based on genetic correlations from the ME model, then these locations can be grouped into a TPE. Regional breeding can begin within this TPE and all multi-location trials are carried out within this TPE. For example, for the traits VIGOR, DM and MCMDS that have correlated responses to selection ranging from 66 to 87% (Table 4), Ubiaja and Ibadan can belong to same TPE.

### Parameter estimates and implications for cassava breeding

The estimates of genomic correlations and heritabilities in Table 5 have interesting implications for cassava genetic improvement. Genetic correlations between RTNO and FYLD estimated with the MT model were high and positive for all locations (ranging from 0.65 to 0.8), whereas those between RTNO and DM and between FYLD and DM were close to zero (ranging from − 0.02 to 0.20). The genetic correlations for these core production traits (DM, RTNO and FYLD) indicate that concurrent improvement of these traits is achievable. However, more replication in trials targeting these production traits will help reduce error variances and improve the accuracy of parental selections given the low heritabilities for FYLD and RTNO. VIGOR can also be improved concurrently with these production traits since it is mostly positively correlated with these (Table 5). The disease trait (MCMDS) showed moderate to strong negative genomic correlations with VIGOR and the production traits, which is favorable for cassava breeding in Africa especially where the cassava mosaic disease (CMD) pressure is high. Consequently, cassava breeders have selected for CMD resistance genes over time [57, 58]. With the favorable genetic correlations between these target traits in mind, the merit index from MT breeding values should be efficient since it takes genetic correlations into account.

**Table 5:** Genetic correlations and heritabilities for the traits analyzed

Trait	VIGOR	RTNO	DM	MCMDS	FYLD	MCGM
Ubiaja						
VIGOR	*0.16*					
RTNO	0.63 (0.01)	*0.21*				
DM	0.27 (0.01)	0.19 (0.01)	*0.42*			
MCMDS	− 0.67 (0.02)	− 0.53 (0.01)	− 0.22 (0.03)	*0.62*		
FYLD	0.62 (0.01)	0.80 (0.01)	0.11 (0.01)	− 0.42 (0.02)	*0.26*	
MCGM	0.05 (0.01)	− 0.03 (0.00)	− 0.17 (0.01)	0.22 (0.01)	− 0.08 (0.01)	*0.1*
Mokwa						
VIGOR	*0.06*					
RTNO	− 0.11 (0.01)	*0.16*				
DM	0.12 (0.02)	− 0.003 (0.01)	*0.31*			
MCMDS	− 0.03 (0.02)	− 0.35 (0.01)	− 0.14 (0.02)	*0.64*		
FYLD	0.04 (0.01)	0.65 (0.01)	− 0.15 (0.01)	− 0.18 (0.01)	*0.21*	
MCGM	0.32 (0.01)	− 0.15 (0.01)	− 0.02 (0.01)	− 0.03 (0.01)	− 0.1(0.01)	*0.26*
Ibadan						
VIGOR	*0.19*					
RTNO	0.46 (0.01)	*0.26*				
DM	0.18 (0.02)	0.20 (0.02)	*0.37*			
MCMDS	− 0.64 (0.03)	− 0.52 (0.03)	− 0.13 (0.03)	*0.77*		
FYLD	0.34 (0.02)	0.77 (0.02)	− 0.02 (0.02)	− 0.44 (0.04)	*0.35*	
MCGM	− 0.14 (0.01)	0.16 (0.01)	− 0.08 (0.01)	0.11 (0.03)	0.11 (0.02)	*0.22*

Plot-based heritabilities on the diagonal, genetic correlations from the MT model on the off-diagonal (standard error of estimates in brackets)

*VIGOR* seedling vigor, *RTNO* number of storage roots per plot at harvest, *FYLD* fresh weight of harvested roots in tons per hectare, *DM* percentage dry matter of storage roots, *MCMDS* plot mean cassava mosaic disease severity, *MCGM* plot mean cassava green mites severity

We would like to make it clear here that fitting MT and ME models are computationally expensive since, in our case, they required the estimation of 90 and 36 additional covariance parameters for the MT and ME models, respectively, compared to the uT and uE models. We had a few thousand records to estimate these parameters accurately for our target traits as shown by the standard errors of these estimates in Tables 4 and 5. When these correlations are not significant, breeding values from univariate models are sufficient because MT models are not expected to result in improved prediction accuracies [59].

## Conclusions

The effectiveness of a breeding program is evaluated by its ability to provide adapted and productive varieties to the farming community in the target environments it serves. To achieve this goal for the cassava breeding program at IITA, we recommend a decentralized breeding strategy for the different agro-ecological zones in Nigeria using total merit indices based on MT breeding values. Further studies should be conducted to understand how much selection gain can be achieved by using this strategy. ME models provided less improvement in prediction accuracy but were useful for understanding GxE interactions.
